# Activation volume of selected liquid crystals in the density scaling regime

**DOI:** 10.1038/srep42174

**Published:** 2017-02-09

**Authors:** A. Grzybowski, S. Urban, S. Mroz, M. Paluch

**Affiliations:** 1 Institute of Physics, University of Silesia in Katowice, ul. Uniwersytecka 4, 40-007 Katowice, Poland; 2 Silesian Center for Education and Interdisciplinary Research, ul. 75 Pułku Piechoty 1a, 41-500 Chorzów, Poland; 3 Institute of Physics, Jagiellonian University, S. Łojasiewicza 11, 30-348 Kraków, Poland

## Abstract

In this paper, we demonstrate and thoroughly analyze the activation volumetric properties of selected liquid crystals in the nematic and crystalline E phases in comparison with those reported for glass-forming liquids. In the analysis, we have employed and evaluated two entropic models (based on either total or configurational entropies) to describe the longitudinal relaxation times of the liquid crystals in the density scaling regime. In this study, we have also exploited two equations of state: volumetric and activation volumetric ones. As a result, we have established that the activation volumetric properties of the selected liquid crystals are quite opposite to such typical properties of glass-forming materials, i.e., the activation volume decreases and the isothermal bulk modulus increases when a liquid crystal is isothermally compressed. Using the model based on the configurational entropy, we suggest that the increasing pressure dependences of the activation volume in isothermal conditions and the negative curvature of the pressure dependences of isothermal longitudinal relaxation times can be related to the formation of antiparallel doublets in the examined liquid crystals. A similar pressure effect on relaxation dynamics may be also observed for other material groups in case of systems, the molecules of which form some supramolecular structures.

Liquid crystalline (LC) phases are formed by molecules having strongly anisotropic shapes. In the present case the elongated (rod-like) molecular systems are considered. Depending upon the chemical structure of the rigid molecular core and two terminal groups, such substances can form the nematic (N) phase with long-range orientational order of the symmetry molecular axes, and/or the smectic phases, showing besides the nematiclike orientational order, some degree of translational order (Sm A and Sm C with a translational order in one dimension and Sm B, I, F, L with an additional bond-orientational long-range order) as well as several crystalline phases classified as hexagonal in-plane lattice (B, J, G, M) and rectangular in-plane lattice (E, K, H, N)^1^.

In the last twenty five years, numerous organic substances forming liquid crystalline (LC) phases were intensively studied under elevated pressure (see reviews refs 2 and 3). Basically, three experimental methods were employed that elucidated different physical properties of the examined substances. Differential thermal analysis (DTA), supported some times by the polarizing optical microscopy (POM) observations, enabled determination of the p-T phase diagrams. Dielectric spectroscopy (DS) measurements yielded information about the molecular dynamics in the LC phase as functions of temperature and pressure^2^,^3^,^4^,^5^,^6^. Pressure-volume-temperature (pVT) studies of selected substances were the basis for a deep analyses of the observed phenomena under different thermodynamic conditions: isobaric, isothermal and isochoric. For example, it was found that the slope of the T(p) clearing line (LC phase – isotropic liquid transition) depends on the details of the molecular structure whereas the contribution of the configuration entropy to the total entropy change depends upon the type of the LC phase^3^.

A main part of high pressure investigations of LCs has been devoted to study the molecular rotations around the short axes in different LC phases. The corresponding low-frequency relaxation time τ_ll_ of this process can be parameterized in terms of activation quantities:

activation enthalpy:


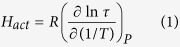


activation energy:


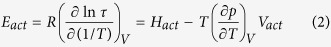


activation volume:


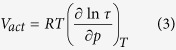


It should be noted that the activation quantities were suggested by Williams^7^ a half century ago to investigate the dielectric relaxation in different thermodynamic conditions by adopting the Eyring transition state theory^8^,^9^ to characterize the thermodynamic evolution of the characteristic timescales (called relaxation times τ) for different dielectric relaxation processes. The relation derived by Eyring for the rate *k* at which species relax between two nonactivated states via an activated one has been successfully applied by Williams to the dielectric relaxation time (*τ* = 1/*k*). Nevertheless, it is worth mentioning that the considered dielectric relaxation is in general not any relaxation of the order parameter to its equilibrium value near a second-order phase transition, especially in case of the liquid-glass transition, which falls outside the thermodynamic classification of phase transitions due to its kinetic character recently expressed by some of us via a universal equation for the pressure coefficient of the glass transition temperature *dT*_*g*_/*dp*^10^. The concept of the dielectric relaxation time has been defined by Debye for polar liquids as the time required for dipole moments of molecules to revert to a random distribution after removal of the applied field^11^. In given thermodynamic conditions, the dielectric relaxation process considered by Debye is characterized by a single relaxation time τ_D_ and a relatively narrow and symmetric dielectric loss peak in the frequency domain. Most dielectric relaxation processes observed experimentally (including those analyzed here for liquid crystal and glass forming systems) exhibit dielectric relaxation loss peaks that are broadened (symmetrically or asymmetrically) in comparison with the Debye dielectric relaxation process and often represented as a superposition of the Debye processes with different relaxation times τ_D_ at given thermodynamic conditions (*T, p*). It means that a distribution of microscopic relaxation times is mostly assumed for a non-Debye relaxation process as has been for instance suggested by Powles in context of discussing the effect of a few internal fields on the dielectric relaxation time and the dielectric spectrum^12^. Using experimental data, the estimated macroscopic dielectric relaxation time τ of such a non-Debye dielectric relaxation process is usually determined from the frequency *f*_*max*_ at the maximum of the measured dielectric relaxation loss peak, i.e., *τ* = 1/(*2πf*_*max*_). Then, the meaning of the activation quantities defined by Eqs (1, 2, 3) can be discussed in terms of a generalized Arrhenius law for different thermodynamic conditions, *τ(T, p*) = *τ*_0_exp[(*H*_*act*_ + *pV*_*act*_)*R*^−1^*T*^−1^], shown here for the temperature-pressure domain, where *H*_*act*_ and *V*_*act*_ can depend on thermodynamic conditions^7^. Thus, *H*_*act*_ is a macroscopic apparent activation enthalpy reflecting a change in enthalpy, which is needed by a dipolar entity to reorient between two local energy minima at *p* = *const, E*_*act*_ is an analogous macroscopic apparent activation energy at *V* = *const*, and *V*_*act*_ is a macroscopic apparent activation volume reflecting a free surrounding space needed by a dipolar entity to reorient between two local energy minima at *T* = *const*. Therefore, the activation volume is a key parameter that characterizes the isothermal pressure dependences of the dielectric relaxation times, which are analyzed in this paper.

All the activation quantities have been found to be very useful to study the rapid increase in the structural relaxation time τ of glass forming (GF) systems, which is observed when a GF liquid is approaching the glass transition. This phenomenon is called the super-Arrhenius behavior due to increasing values of *H*_*act*_ and *V*_*act*_ under isobaric cooling and isothermal compression, respectively^13^. For many nematics, smectics, and the crystalline E phase (Cr E) that reveals certain orientational degrees of freedom besides the long-range three-dimensional crystalline order, the activation energy is roughly half the value of the activation enthalpy, whereas the activation volume is ca. 20% of the molar volume. Thus, LC systems show many similarities with the behavior of the structural relaxation times in the glass forming (GF) systems. Unfortunately, the theoretical basis for discussion of the dynamic properties of molecules in LC phases is rather poor, in contrast to GFs. Therefore, our analyses of the thermodynamic aspects of the τ_||_(pVT) behaviors have been based on the power density scaling (PDS) law, which was originally formulated for GF systems and successfully verified for many material groups of GFs, but also satisfactorily applied to describe the thermodynamic evolution of the longitudinal relaxation times of various LC systems in different LC phases^3^,^6^,^14^,^15^,^16^,^17^,^18^,^19^. In this context, it is worth noting that there are two significant differences between the LC and GF systems: (i) the range of T and p in which a LC phase is thermodynamically stable is rather limited, in comparison with the supercooled regime of GF systems; (ii) there is no experimental evidence for non-Arrhenius temperature-dependences of τ_||_ within LC phases, whereas such behavior is a common feature of GF substances. In addition, it should be borne in mind that the number of LC materials studied at elevated pressure is rather limited (about 20), whereas hundreds of GF materials have been investigated at high P. It was found that τ_||_ determined for many LCs forming different phases (nematic, smectic A, smectic C, crystalline E, cholesteric) can be successfully scaled to a master curve like in the GF systems as a function of a single scaling variable, *ρ*^*γ*^/*T*, where ρ is the material density and the scaling exponent γ is a material constant, which may depend on LC phases of a given LC system^3^,^6^,^7^,^8^,^9^,^10^,^11^,^12^,^13^,^14^,^15^,^16^,^18^,^19^.

It should be emphasized that the power law density scaling has been acknowledged as a promising unifying idea in the glass transition physics^20^ and successfully used to differentiate between physically relevant and irrelevant models of the thermodynamic evolution of the characteristic timescale of molecular dynamics in the GF systems^21^. Since the case of LC systems has shown that the applicability of the density scaling law goes beyond the GF systems, a comparative study of LC and GF systems in the density scaling regime are highly needed to give a better insight into their universal description.

In this paper, we concentrate our attention on the analysis of the activation volume in the density scaling regime taking into account the theoretical models recently developed and checked for GF systems^22^,^23^. Nevertheless, our motivation of this work is two-fold: (i) to thoroughly investigate unusual pressure effects on molecular dynamics of LC systems, which are opposite to those observed for GF systems, and (ii) to verify whether leading entropic models formulated in the power law density scaling limit from Avramov and Adam-Gibbs models, which are based respectively on the total and configurational entropies, can be also employed in the investigations of LC systems similarly to their successful applications to study GF systems. Consequently, we shed a new light on the fervently discussed problem in condensed matter physics and material engineering sciences as to *how to properly and most universally model the thermodynamic evolution of the timescale of molecular dynamics in different materials*.

## Theoretical models

The activation volume defined by Eq. (3) is a fundamental characteristic of materials subjected to squeezing, which naturally informs us about the sensitivity of molecular dynamics of the materials to changes in pressure under their isothermal compression. In general, values of the parameter V_act_ depend on thermodynamic pVT conditions and can be expressed for instance as a temperature-pressure function V_act_(T, p). However, high pressure experimental data are usually measured too rarely to accurately determine such a function V_act_(T, p) in a direct way. Therefore, we need to exploit some models to describe the experimental dependence τ_||_(T, P), and then reliably evaluate the function V_act_(T, p).

As already mentioned, both GF and LC systems have been successfully studied in terms of the thermodynamic scaling idea, which provides us an attractive way to relate macroscopic experimental dynamic quantities (such as diffusivity, viscosity, structural relaxation time, segmental relaxation time in polymers, and longitudinal relaxation times in LCs) to an effective intermolecular potential, 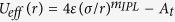, which includes a small attractive background *A*_*t*_ and a dominant repulsive inverse power law (IPL) term with *m*_*IPL*_ ≈ 3*γ*, where γ is the scaling exponent of the scaling variable *ρ*^*γ*^/*T* for a given material. The simple soft-sphere potential discussed to be valid for viscous liquids and disordered solids highly inspired a development in the condensed matter physics. In the last decade, a few models have been suggested^23^,^24^,^25^,^26^,^27^,^28^ to describe the temperature-density dependences of the dynamic quantities, *Y(T, ρ*), which obey the power law density scaling law, *Y* = *f(ρ*^*γ*^/*T*). Among them, the temperature-volume versions of Avramov^26^,^27^ and MYEGA^23^ models have attracted a lot of interest due to their entropic fundamentals, especially that the Avramov model is based on the total system entropy *S*^26^,^27^,^29^,^30^,^31^ while the MYEGA model has been derived^23^,^32^ from the well-known Adam-Gibbs model^33^, which relies on the configurational entropy *S*_*c*_ recently shown^21^ to obey the PDS law with the same value of the scaling exponent γ that scales structural relaxation times of GF systems. Therefore, we have considered both the models in our investigations of the activation volume of LC systems,

T-V Avramov:


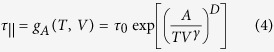


T-V MYEGA:


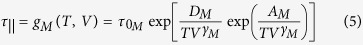


where the specific volume *V* = *ρ*^−1^ and *τ*_0_, *γ, A, D* are fitting parameters of the T-V Avramov model and their symbol counterparts with the subscript M are fitting parameters of the T-V MYEGA model, respectively.

Since the PDS law, *Y* = *f(ρ*^*γ*^/*T*), can be extended to a more general form^25^,^34^, *Y* = *g*_mod_(Γ), where the scaling variable, Γ = *h(ρ*)/*T*, can be expressed by a density function *h(ρ*) which is not limited to the power density function *h(ρ*) = *ρ*^*γ*^ with a constant scaling exponent γ, we discuss the activation volume of a dynamic quantity given by the function *Y* = *g*_mod_(Γ). Then, Eq. (3) can be represented as follows, 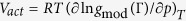, and separated into two terms 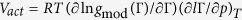, where *RT*(∂*l*Γ/∂*p*)_*T*_ is independent of the assumed model *Y* = *g*_mod_(Γ) and 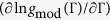 is the model-dependent derivative. The former term *RT*(∂*l*Γ/∂*p*)_*T*_ = *RTγ*Γ/*B*_*T*_(*p*), where 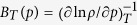 is the isothermal bulk modulus for volume and the scaling exponent 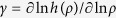 is defined in a general density-dependent form^35^, which can be reduced to a constant parameter in the PDS law limit, i.e., if *h(ρ*) = *ρ*^*γ*^ with *γ* = *const*. Thus, one can derive the following general formula for the activation volume in the general density scaling (GDS) regime


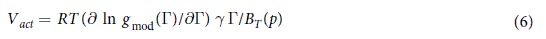


If we consider the T-V Avramov and T-V MYEGA models, *τ*_||_ = *g*_*A*_(Γ) and *τ*_||_ = *g*_*M*_(Γ), given by Eqs (4) and (5), respectively, the model-dependent contributions 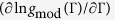 to Eq. (6) will be as follows


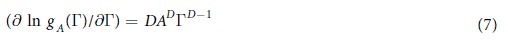



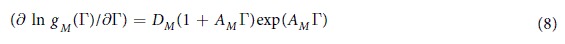


A few years ago, an equation of state (EOS) was derived^22^ to facilitate analyses of the activation volumetric properties, e.g., the isothermal bulk modulus for the activation volume defined by analogy with *B*_*T*_(*p*) as 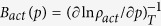, where 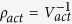. In the activation volumetric EOS,


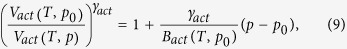


two temperature parametrization have been used


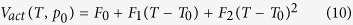



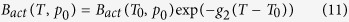


where *F*_0_ = *V*_*act*_(*p*_0_, *T*_0_), 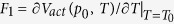, 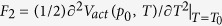, and 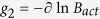 
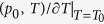
. Equation (9) has been well interpreted in terms of the thermodynamic scaling idea^22^, because this activation volumetric EOS has been patterned on an volumetric EOS earlier derived^36^,^37^ in the PDS regime,


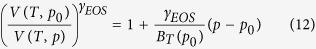


on the basis of another EOS suggested^38^ for a low compression region. Equation (12) has been later parametrized by the following two temperature functions^39^,


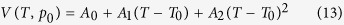



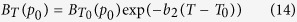


where *A*_0_ = *V*_0_(*T*_0_) = *υ(p*_0_, *T*_0_), 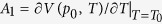, and 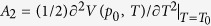, 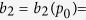 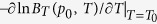.

It should be stressed that Eqs (9) and (12) can be also derived^22^,^37^,^40^ in the ways independent of the thermodynamic scaling idea. This possibility extends the applicability range of the EOSs to materials, the molecular dynamics of which does not obey the PDS law. One of the alternative derivation ways relies on the assumption that the appropriate isothermal bulk moduli linearly depend on pressure as follows


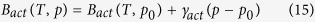



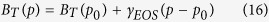


where *γ*_*act*_ = (∂*B*_*act*_(*p, T*)/∂*p*)_*T*_ = *const* and *γ*_*EOS*_ = (∂*B*_*T*_(*p*)/∂*p*)_*T*_ = *const*. Moreover, it is worth noting that Eqs (9) and (12) imply Eqs (15) and (16), respectively.

In the case of the supercooled state of GF systems, the reference state (T_0_, P_0_) for the activation volumetric and volumetric EOSs is usually chosen at the glass transition temperature at ambient pressure. Nevertheless, both the EOSs can be used in other thermodynamic phases, including LC phases, if their activation volumetric and volumetric properties vary in the applicability ranges of the equations, which are defined by Eqs (10), (11), (15) and (13), (14), (16), respectively^22^,^39^,^41^,^42^,^43^,^44^. By analogy with GF systems, the reference states (T_0_, P_0_) have been fixed for examined LC systems at appropriate LC phase transition temperatures at ambient pressure.

As verified in several papers^22^,^28^,^36^,^37^,^38^,^39^,^40^,^45^,^46^,^47^,^48^,^49^, in case of GF systems that belong to different material groups such as van der Waals liquids, ionic liquids, polymers, associated liquids, and the Kob-Andersen binary Lennard-Jones (KABLJ) liquid, which is a prototypical model of supercooled liquids, the volumetric and activation volumetric EOSs successfully describe the dependences V(T, P) and V_act_(T, P), respectively. Moreover, useful relationships have been formulated^22^ between some parameters of Eqs (9) and (12) on the additional assumption that the activation volume defined by Eq. (3) is well described in terms of the T-V Avramov model (Eq. (4)), which implies that V_act_ is expressed by Eq. (6) with Eq. (7). As a result, it has been found that the pressure derivative of B_act_ and the value of B_act_ at the reference pressure at a given temperature can be determined with no necessity of the activation volume calculations as follows


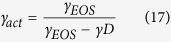



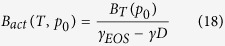


It is worth noting that the above equations are model-dependent due to the parameter D of the T-V Avramov model. In Supplementary Information (Section S1), we have derived their counterparts, which are based on the T-V MYEGA model (Eq. (5)) and also dependent on the model parameter A_M_,


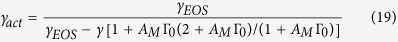



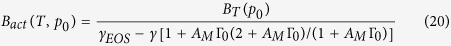


where Γ_0_ = *T*^−1^*V*^−*γ*^(*T, p*_0_) is constant at *T* = *const*. Since one can expect similarly to γ_EOS_ that *γ*_*act*_ = (∂*B*_*act*_(*p, T*)/∂*p*)_*T*_ ≅ *const* at different temperatures, the value of the parameter Γ_0_ should remain unchanged for a given material at least to a good approximation.

## Results and Discussion

We have selected to analyze the dielectric and pVT data for six LC substances that exhibit LC phases in relatively broad (p, T) ranges, such as 4-heptyl-4′-cyanobiphenyl (7CB), trans-4-heptyl(4-cyanophenyl)cyclohexane (7PCH), trans-4-n-octyl-(4-cyanophenyl)cyclohexane (8PCH), n-octyloxy-cyanobiphenyl (8OCB), n-hexyl-isothiocyanato-biphenyl (6BT), and n-octyl-isothiocyanato-biphenyl (8BT). All compounds have strong dipole moments directed along the symmetry axes, which are created by the CN or NCS group attached at one terminal position (at the other the alkyl or alkoxy group is attached). For this reason, the dielectric relaxation spectra yield solely the low frequency relaxation time connected with the flip-flop molecular motions in a given LC phase (N for 7CB, 7PCH, 8PCH and 8OCB, Cr E for 6BT and 8BT). It should be added that the compounds with the CN group show a tendency to forming antiparallel doublets in the LC phases^50^ The experimental dielectric and pVT data examined by us herein have been earlier measured and reported for the nematic phase of 7CB^51^,^52^, 7PCH^53^,^54^, 8PCH^55^,^56^, and 8OCB^18^,^57^ as well as the crystalline E phase of 6BT^58^,^59^ and 8BT^60^,^61^.

**Figure 1:**
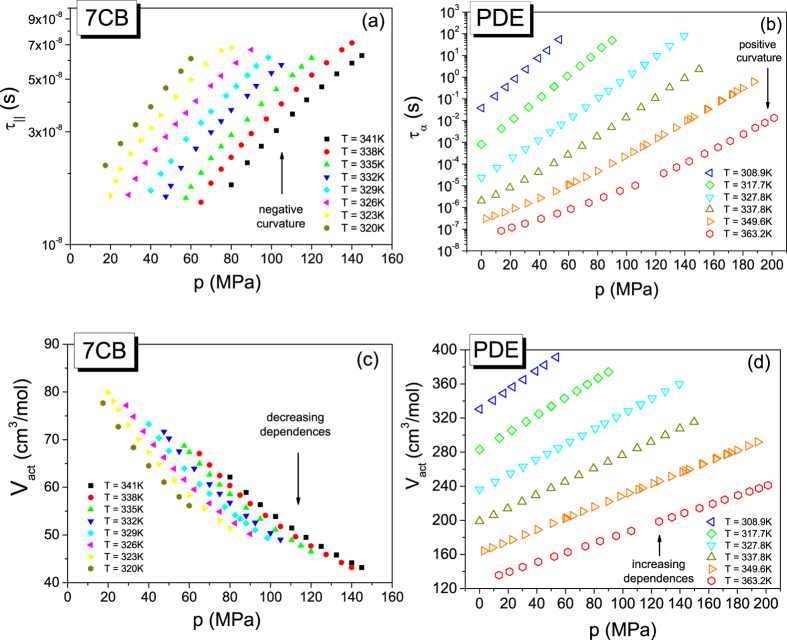
Comparison of the isothermal pressure dependences of longitudinal dielectric relaxation times τ_||_ for a LC system 7CB (**a**) and structural relaxation times τ_α_ for a typical GL system PDE (**b**) as well as the isothermal pressure dependences of the corresponding activation volumes V_act_ for 7CB (**c**) and PDE (**d**). The values of V_act_ for PDE have been reported in ref. 22, whereas the values of V_act_ for 7CB have been calculated herein in the way suggested in ref. 22.

From the inspection of the isothermal pressure dependences of the longitudinal dielectric relaxation times τ_||_ of the LC systems subjected to compression, one can spot an intriguing behavior of the isothermal dependences τ_||_ (p), which increase with increasing pressure as those established for the GF systems but reveal an untypical curvature that is negative in contrast to the positive one commonly observed for the GF systems. For comparison, in Fig. 1, we present the pressure dependences of the longitudinal relaxation times log_10_τ_||_ (p) and the structural relaxation times log_10_τ (p), which have been determined from dielectric measurements on one of the investigated LC system (7CB) and a supercooled GF system (phenolphtalein dimethyl ether (PDE) that is a typical van der Waals liquid) in high pressure isothermal conditions as well as the corresponding activation volumes for the dielectric relaxation times. At first glance, one can see that the pressure isothermal dependences V_act_(p) of PDE increase with increasing pressure, which is the typical behavior of GF systems^22^. However, the isothermal dependences V_act_(p) of 7CB behave quite opposite, i.e., they decrease with increasing pressure.

We apply the formalism outlined in the previous section to analyze the activation volumetric properties of the selected LC systems, because the activation volume V_act_ defined by Eq. (3) and the isothermal bulk modulus for the activation volume, 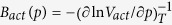, are the macroscopic quantities, which enable us to gain a better insight into the sensitivity of the molecular dynamics of the investigated LC systems to changes in pressure in isothermal conditions. In order to evaluate activation volumes of the investigated LC systems, we first follow the procedure based on the T-V Avramov model (Eq. (4)), which has been earlier successfully applied in ref. 22 to test the activation volumetric EOS (given herein by Eq. (9) with Eqs (11) and (12)) by using experimental data of different GF systems. The procedure relies on the density scaling law, which is obeyed by the longitudinal relaxation times τ_||_ of many LC systems in different LC phases as already mentioned in Introduction. Since dielectric measurements are typically carried out in isobaric and isothermal conditions, we can determine temperature-pressure dependences τ_||_(T, p) from such experiments. To transform the dependences τ_||_(T, p) to their temperature-volume representation τ_||_(T, V), we exploit the experimental pVT data V(T, p), which are parametrized by means of the EOS given by Eq. (10). Then, we can describe the dependence τ_||_(T, V(T, p)) by using the T-V Avramov model (Eq. (4)), the parameters of which enable us to determine the activation volumes from Eq. (6) with Eq. (7). Since the procedure for fitting the dependences τ_||_(T, p) to the T-V Avramov model (Eq. (4)) with V(T, p) taken from the EOS given by Eq. (12) with Eqs (13) and (14) has turned out to be very or even extremely time-consuming due to its very slow convergence at a typical required fit tolerance (
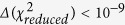
) for most of the investigated LC systems, we present details of such an analysis based on the Avramov model in Supplementary Information (Section S2), using 8BT in the crystalline E phase as an example.

**Figure 2:**
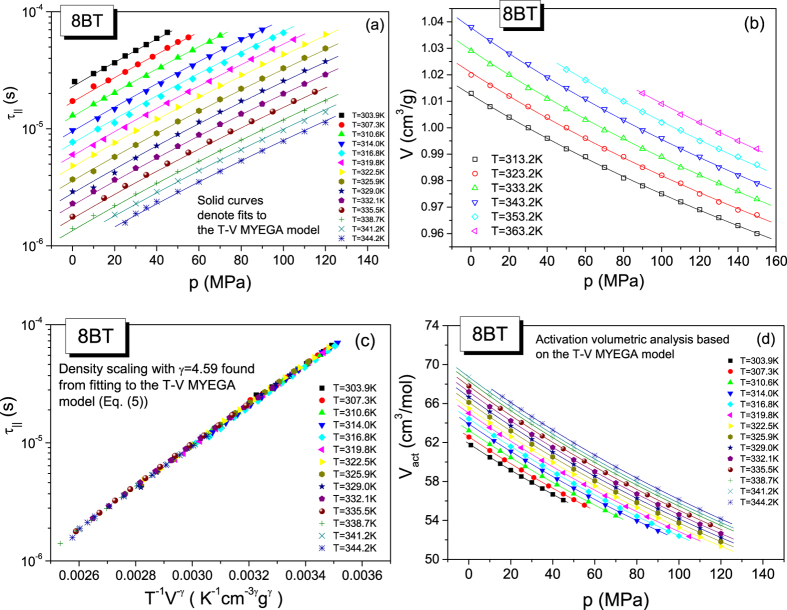
The analysis of the activation volumes of 8BT in terms of the T-V MYEGA model. (**a**) Pressure dependences of experimental longitudinal relaxation times along different isotherms and their fits to Eq. (5) (see Table 2 for the values of its parameters) with the specific volume expressed as the function V(T, p) by the volumetric EOS given by Eq. (12) with Eqs (13) and (14). (**b**) Pressure dependences of specific volumes measured along different isotherms and their fits to Eq. (10) (see Table 1 for the values of its parameters). (**c**) Plot of the master plot according to the power law density scaling law obeyed by longitudinal relaxation times. (**d**) Pressure dependences of the activation volumes evaluated from Eq. (6) with Eq. (8) and their fits to to the activation volumetric EOS given by Eq. (9) with Eqs (10) and (11) (see Table 3 for the values of its parameters).

However, to establish the activation volumes from Eq. (6) with Eq. (8), we need to find the values of the parameters of the MYEGA model by fitting the dependence τ_||_(T, V(T, p)) to Eq. (5). Using also 8BT in the crystalline E phase as an example, we illustrate steps of the analysis in Fig. 2. The dielectric isotherms of 8BT (Fig. 2(a)) measured as a function τ_||_(T, p) have been transformed to its T-V representation (not shown herein) by using the volumetric EOS (Eq. (12) with Eqs (13) and (14)) fitted to the pVT experimental data for 8BT (Fig. 2(b)), making the assumption that the reference state (T_0_, p_0_) in the volumetric EOS is fixed at the crystal – Cr E transition temperature *T*_*0*_ = 301.5 K at ambient pressure *p*_*0*_ = 0.1 MPa. Then, we can directly describe the dependence τ_||_(T, p) as shown in Fig. 2(a) by using Eq. (5) with the specific volume V(T, p) expressed by Eq. (12) with Eqs (13) and (14), where the values of the EOS parameters are found by fitting pVT measurement data to this EOS (see Fig. 2(b) and Table 1). Then, we can successfully use the value of the scaling exponent, γ = 4.59 ± 0.03, evaluated by fitting to the MYEGA model (Eq. (5)) to perform the density scaling of the longitudinal relaxation times of 8BT (see Fig. 2(c)). Subsequently, the isothermal dependences V_act_ can be established for 8BT from Eq. (6) with Eq. (8) at temperatures and pressures (T, p) at which the dependences τ_||_(T, p) have been determined. As shown in Fig. 2(d), we have also fitted the isothermal dependences V_act_ based on the MYEGA model for 8BT to the activation volumetric EOS (Eq. (9) with Eqs (10) and (11)), taking the same reference state (T_0_, p_0_) as that assumed in the previous volumetric analysis performed for this LC system in terms of the volumetric EOS. The same reference state has been used for 8BT in the Cr E phase in the analysis based on the Avramov model (see Supplementary Information). The values of the parameters of Eq. (5) are collected in Table 2 for all tested LC systems, whereas the values of the parameters of the activation volumetric EOS determined on the basis of Eq. (5) are listed in Table 3. However, the values of the parameters of Eq. (4) and the activation volumetric EOS determined on the basis of Eq. (4) are collected in Supplementary Information (in Tables S1 and S2, respectively).

From the comparative studies, it should be noted that the scaling exponents established by using MYEGA and Avramov models, respectively γ_M_ and γ, are the same for a given LC system studied in the Cr E phase and they are mutually in accord within their determination uncertainties for a given LC system in the nematic phase (see Table 2 in the main part and Table S1 in Supplementary Information), which lead to the density scaling of the longitudinal relaxation times of each tested LC system to a good approximation (see Fig. 2(c) for 8BT in the Cr E phase as an example). In addition, one can observe an increase in the value of the scaling exponent with increasing the molecular size of the LC systems, which suggests that the role of repulsive interactions in the molecular dynamics grows if the LC system molecular size is larger. After that general conclusion, we need to note that the MYEGA model has been found by us to be definitely faster convergent at the same fit tolerance as that assumed in case of the Avramov model. We have established that the MYEGA model characterized by the double exponential form also better reflects the negative curvature of the isothermal dependences τ_||_(p) of the examined LC systems (see details of the analysis in Section S3 in Supplementary Information). Thus, we need to look deeper into what determines the activation volume in both the Avramov and MYEGA models.

The analysis presented in Fig. S3 in Supplementary Information suggests that the activation volumes evaluated for a given material from the Avramov and MYEGA model can be in general different, because the curvatures established from these models for the dependence log_10_τ_||_(Γ) can slightly differ for this material (as shown in case of the tested LC systems in the nematic phase). To verify this preliminary conjecture, it is convenient to examine the analytical general equation (Eq. (6)) derived for the activation volume in the GDS regime and its representations based on Eqs (4) and (5), which lead to the special cases of Eq. (6) that involve Eqs (7) and (8), respectively. Since the model-dependent and model-independent terms are well separated in the general equation (Eq. (6)), we can analyze both the contributions to the activation volume in case of the Avramov and MYEGA models. The model-independent term, *RTγ*Γ/*B*_*T*_(*p*), combines volumetric and effective short-range intermolecular potential properties, involving the isothermal bulk modulus for the specific volume *B*_*T*_(*p*) and the scaling exponent γ, which is a material constant in the PDS regime that enables us to describe the molecular dynamics in terms of the scaling quantity Γ = *T*^−1^*V*^−γ^. As already mentioned, the values of scaling exponents obtained in the Avramov and MYEGA models for each material are almost the same. Thus, the activation volumes evaluated using these models would have been also nearly the same for a given material if the model-dependent term had quantitatively contributed to Eq. (6) on the same level in case of both the models for the material. From Eqs (7) and (8), one can see that the model-dependent contributions to Eq. (6) are some functions of the scaling variable Γ, which are parametrized by the parameters A and D as well as A_M_ and D_M_ of the Avramov and MYEGA models, respectively. It confirms that the activation volume values evaluated for a given material by using these models in the PDS regime (Eqs (4) and (5)) in general depend not only on the density scaling exponent and volumetric properties for this material.

We have evaluated and compared the activation volumes evaluated by using the T-V MYEGA and Avramov models for each examined LC system. As an example, we have presented the plots of the dependences V_act_(p) for 8BT in Fig. 2(d) in the main part and S1(d) in Supplementary Information, respectively. From these figures and other such data not shown herein, we can claim that both the models yield slightly different values of the activation volume despite almost the same values of the scaling exponent γ_M_ and γ for a given material. The differences are related to the model-dependent contributions (Eqs (8) and (7)) to the activation volume defined in the GDS regime by Eq. (6). Thus, it is worth discussing the other parameters of Eqs (8) and (7) than the scaling exponent, i.e., the parameters A_M_ and D_M_ as well as A and D, respectively. First of all, it should be stressed that the fitted values of the parameters found for the investigated LC systems (see Table 2 for the MYEGA model parameters A_M_ and D_M_ and Table S1 in Supplementary Information for the Avramov model parameters A and D) show significant differences in comparison with those earlier widely reported^22^,^23^,^26^,^27^,^62^,^63^ for many GF systems. Striking differences have been established in case of the parameters A and D of the Avramov model. The values of the former are extremely large, especially for the LC systems investigated in the nematic phase, whereas the values of the parameter D ranges between 0 and 1, which have never been observed yet in other material groups and requires considering its physical meaning for the LC materials. Since the parameter *D* of the Avramov model has been interpreted to be inversely proportional to the number of available pathways for local motions of a molecule or polymer segment^26^,^29^ (as also mentioned in Section S4 in Supplementary Information), we need to doubt the possibility that there are sufficiently many such pathways in the LC systems to cause the value of the parameter D < 1. On the other hand, the values of the parameter D_M_ of the MYEGA model are at most an order of magnitude greater than those reported for the GF systems, whereas the values of the parameter A_M_ are surprisingly negative for each tested LC system.

Taking into account the fundamentals of the MYEGA model^32^,^23^ (which considers the network constraints using a two-state pattern according to that they are either intact or broken with the energy difference represented by the parameter A_M_), we can suggest that the negative values of this parameter obtained for the investigated LC systems can be related to forming antiparallel doublets in these materials as already mentioned. The doublet formation energy can be considered similarly to the dimerization energy that is usually negative as a difference between the energy of a dimer, which is an energetically favorable state, and the sum of the energy of two monomers. Similarly to the interaction between molecules forming dimers^64^, the negative energy of the intermolecular interaction causing two molecules to form an antiparallel doublet is expected to be accompanied by the negative entropy of this interaction. The T-V MYEGA model is capable to satisfactorily describe such a case, because the configurational entropy originally considered in this model for GF systems can be adopted to represent the negative entropy of the interaction between two LC molecules forming the antiparallel doublet. This is because the entropy of such interactions can be reinterpreted within the T-V MYEGA model for LC systems in terms of Eq. (S21) earlier postulated^23^ for GF liquids in the way outlined in Section S4 in Supplementary Information. In the MYEGA model, the number of floppy modes has been considered as the number of broken constraints^65^,^66^. If we assume that the intact constraints result in forming the antiparallel doublets, the breakage of such a constraint leading to two unconstrained states (molecules) requires providing a sufficient energy to break the interaction between the molecules forming the antiparallel doublet. Thus, the floppy mode considered as a broken antiparallel doublet involves two unconstrained states (molecules) characterized by the same energy of the breakage of the antiparallel doublet. This simple picture shows that the degeneracy of the floppy mode can be quantified by Ω = 1/2 in terms of the unconstrained state, and then lnΩ is negative, similarly as that evaluated in some cases of recently reported predictions based on the MYEGA model for supercooled borate and silicate liquids^66^. Thus, in case of examined LC systems, the preexponential factor dependent on lnΩ is also negative in Eqs (S21) and (S22) presented in Section S4 in Supplementary Information. Then, Eq. (S22) results in an increase in the interaction entropy (in the domain of negative values) related to forming the antiparallel doublets by molecules of the examined LC systems due to an increase in the interaction energy *H*_*M*_ with increasing density in isothermal conditions, which is predicted by Eq. (S18) earlier postulated^23^ for GF liquids as mentioned in Section S4 in Supplementary Information. However, in case of the tested LC systems, the latter behavior is expected in the domain of negative values following the negative value of the fitting parameter A_M_ of the T-V MYEGA model (Eq. (4)), because the parameter A_M_ involves the reference value of the interaction energy *H*_*M*_(*V*_*r*_) according to Eq. (S20) shown in Section S4 in Supplementary Information. In this way, the interaction entropy increases with increasing the interaction energy, which occurs in the domains of negative values for both the quantities, similarly as often reported on the dependence of entropy on energy for interactions leading to a dimer formation^67^. It is worth noting that the negative values of the interaction entropy are compensated by the negative value of the parameter *B*_*M*_ in the Adam-Gibbs equation^33^ underlying the MYEGA model^23^,^32^, 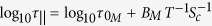. As discussed in Section S4 in Supplementary Information, the parameter *B*_*M*_ depends on the critical configurational entropy 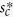. In case of the examined LC systems, the value of the critical entropy 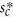 is expected to be negative, because it can be interpreted as the limiting value of the interaction entropy related to the minimal value of the interaction energy. which is required to form antiparallel doublets. The reinterpreted fundamentals of the MYEGA model rationalize our hypothesis that the negative value of the parameter A_M_ of the MYEGA model as well as the negative curvature of the dependences τ_||_(p) can reflect the formation of antiparallel doublets in the examined LC systems.

**Figure 3:**
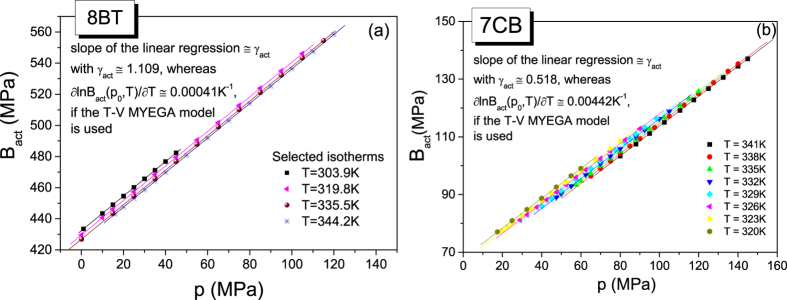
Plot of the pressure dependences of the isothermal bulk moduli for the activation volumes evaluated in terms of the T-V MYEGA model for 8BT in the Cr E phase and 7CB in the nematic phase (panels (**a**) and (**b**), respectively). The solid lines denote the linear dependences that are in accord with Eq. (11), which complies with the activation volumetric EOS given by Eq. (9) with Eqs (10) and (11) (see Table 3 for the values of its parameters).

To gain a better insight into the activation volumetric properties of the LC systems, we have described the pressure dependences of the activation volumes by the activation volumetric EOS. The dependences V_act_(p) have been very successfully fitted to the activation volumetric EOS independent of the model (MYEGA or Avramov) used to evaluate the values of V_act_ (see Fig. 2(d) in the main part and S1(d) in Supplementary Information as examples). Nevertheless, the differences in the values of the activation volumes determined from the MYEGA and Avramov models have been also reflected in the values of the activation volumetric EOS parameters (see Table 3 in the main part and Table S2 in Supplementary Information). It should be noted that the differences in the activation volumes considerably affect the isothermal bulk moduli for the activation volumes (see Fig. 3 in the main part and Fig. S2 in Supplementary Information as examples), and consequently the different activation volumetric properties can be better demonstrated by using the dependences B_act_(p) than V_act_(p). We first need to stress that the values of B_act_ are positive. Such an activation behavior results from the decreasing pressure dependence of the activation volume of the tested LC systems, which is quite opposite to the activation volumetric behavior of the GF systems (for which the dependences V_act_(p) increase and the dependences B_act_(p) decreases with increasing pressure, and the values of B_act_(p) are negative). Thus, the activation volumetric behavior is qualitatively in accord with the typical pressure behavior of the specific volume V and the isothermal bulk modulus B_T_ for the volume. Moreover, all the dependences B_act_(p) increase with increasing pressure in the linear manner according to Eq. (11), which complies with Eq. (9) that very well describes the dependences V_act_(p) determined from both the MYEGA and Avramov models. Nevertheless, the values of the exponent γ_act_ (which is also the slope of the linear dependence B_act_(p)) are less in case of the activation volumes determined from the MYEGA model, and they can be even over 2 times smaller (see Table 3 in the main part and Table S2 in Supplementary Information) than those established for the activation volumes evaluated from the Avramov model. The different slopes of the linear isothermal dependences B_act_(p) can be seen for 8BT and 7CB as examples in Fig. 3 in the main part and Fig. S2 in Supplementary Information, depending on the model used to evaluate the activation volumes. However, the most striking observation can be made from Fig. 3. This is an extremely low sensitivity of the activation volumetric prosperities to changes in temperature at a given pressure in case of the activation volumes determined from the MYEGA model. For this reason, in Fig. 3(a), only some chosen dependences B_act_(p) have been shown in order to avoid making this panel of Fig. 3 obscure. This very weak temperature effect on the isothermal bulk modulus for the activation volume at a given pressure is reflected in small values of the parameter 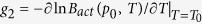 of Eq. (11), which can even reduce to 0 as established for 8PCH and 8OCB in their nematic phase. One can expect that this surprising activation volumetric behavior is also related to some networking of LC systems via the formation of antiparallel doublets.

Finally, we have tested the relations given by Eqs (17) and (19) by using the experimental data for all the studied LC systems and the results of their analyses by employing the T-V Avramov and MYEGA models (Eqs (4) and (5)) as well as the activation volumetric and volumetric EOSs (Eqs (9, 10, 11) and (12, 13, 14)). We have established a very good agreement between the calculated values of γ_act_ from Eq. (17) and those found by fitting the isothermal dependences V_act_(p) to the activation volumetric EOS if the values of V_act_(p) have been evaluated from the Avramov model (Eq. (6) with Eq. (7)). Since the relations given by Eqs (17) and (19) are model-dependent, it has not been able to apply Eq. (17) to satisfactorily predict values of the exponent γ_act_ obtained from fitting the dependences V_act_(p) to the activation volumetric EOS if the values of V_act_(p) have been determined from the MYEGA model. Therefore, we have employed Eq. (19) in this case. As already mentioned in the previous section, Eq. (19) is an approximate equation and requires remaining a value of the scaling quantity Γ_0_ nearly unchanged for a given material. As a consequence, we have needed to find an appropriate value of Γ_0_ for each LC material prior to calculating its exponent γ_act_ from Eq. (15). To successfully predict the value of γ_act_ from Eq. (19), which are in accord with that obtained by fitting the dependences V_act_(p) determined from the MYEGA model to the activation volumetric EOS, we have used the values of Γ_0_ close to the upper experimental limit of the scaling quantity Γ (the experimental ranges of Γ are shown in Fig. S3 in Supplementary Information) in case of the LC systems investigated in the nematic phase, and the values of Γ_0_ about 50% larger than the upper experimental limits of the scaling quantity Γ for 6BT and 8BT in the Cr E phase. It should be noted that the T-V MYEGA model has been applied to evaluate the activation volumes for the first time to our best knowledge, and Eq. (19) is the first attempt made to interrelate the exponents γ, γ_EOS_, and γ_act_, which requires further studies to enhance its prediction capability.

## Summary and Conclusions

In this paper, we have thoroughly investigated the activation volumetric properties of the selected LC systems in different phases (N and Cr E), which obey the power law density scaling law for the longitudinal dielectric relaxation times. Inspired by the negative curvatures of the pressure dependences of the isothermal longitudinal relaxation times τ_||_(p), which is quite opposite to those typically obtained for the structural relaxation times of many GF systems, we have compared the activation volumetric properties of LC and GF systems in the PDS regime. For this purpose, we have applied the formalism based on the temperature-volume versions of the Avramov and MYEGA models (Eqs (4) and (5)), which has been also developed by us herein within the framework of the MYEGA model. Based on the general equation (Eq. (6)) for the activation volume in the general density scaling regime, we have been able to examine the model-independent and model-dependent contributions to V_act_. We have also exploited the activation volumetric and volumetric equations of state (Eqs (9, 10, 11) and (12, 13, 14)) recently derived for the viscous liquids and well interpreted in the PDS regime. The volumetric EOS has enabled us to transform between the sets of thermodynamic variables (T, p) and (T, V), and the activation volumetric EOS has considerably facilitated the analysis of the activation volumetric properties of the tested LC systems.

We have established that the negative curvature of the dependences τ_||_(p) for all the examined LC systems results in the unusual activation volumetric properties that are quite opposite to those widely reported for many GF systems. Among the activation properties of the LC systems, the special attention should be paid to a few of them: (i) the decrease in the activation volume with increasing pressure in isothermal conditions, (ii) the increase in the isothermal bulk modulus for the activation volume with increasing pressure, B_act_(p), which satisfies the linear dependence (Eq. (15)) that complies with the activation volumetric EOS (Eq. (9)) well describing the dependences V_act_(p) in the tested experimental range, (iii) the positive values of B_act_, which are a consequence of the increasing dependence V_act_(p). All the activation volumetric properties are qualitatively in accord with those typically reported for the specific volume and the isothermal bulk modulus for the specific volume of the LC and GF systems, whereas it cannot be said about the typical activation volumetric properties of GF systems. The analysis based on the activation volumetric EOS has additionally revealed a weak sensitivity of the activation properties (for instance B_act_) of the examined LC systems to changes in temperature. The comparative studies of the activation volume for the selected LC systems in terms of the T-V Avramov and MYEGA models lead to the conclusion that the MYEGA model is able to more efficiently describe the dependences τ_||_(p) characterized by the negative curvature and the fitted values of its parameters are more reasonable from the viewpoint of the physical meaning of the model parameters. Within the framework of the MYEGA model, we have achieved a very interesting result, which is the unexpectedly negative value of the parameter A_M_ in Eq. (5) for each tested LC system. The energetic interpretation of this MYEGA model parameter (based on the two-state model of constraints that can be either intact or broken) allows us to suggest that the negative value of the parameter A_M_ is related to the energy barrier for the formation of antiparallel doublets, which most likely occurs in the case of examined LC systems. This finding can be also regarded as an indirect explanation for the negative curvature of the pressure dependences of isothermal longitudinal relaxation times observed for the LC systems as well as its consequence that is the decrease in activation volumes with increasing pressure. Thus, the MYEGA model seems to be a useful tool to explore properties of liquid crystals, which is worthy of confirmation in further investigations of other LC systems. The observed advantage of the MYEGA model over the Avramov model in analyzing relaxation dynamics of selected LC systems is an important outcome of our study, which is a relevant clue in search of the most universal description of the thermodynamic evolution of the timescale of molecular dynamics in different materials.

It should be emphasized that the pressure effect on relaxation dynamics, which is observed in case of LC systems, has an extraordinary character that enhances our understanding of relaxation behaviors, which are possible in complex materials subjected to various thermodynamic conditions. One can expect that representatives of other material groups may also behave at elevated pressure similarly to the examined LC systems, if their molecules form some special supramolecular structures. However, this suggestion should be verified in further studying.

## Additional Information

**How to cite this article**: Grzybowski, A. *et al*. Activation volume of selected liquid crystals in the density scaling regime. *Sci. Rep.* **7**, 42174; doi: 10.1038/srep42174 (2017).

**Publisher's note:** Springer Nature remains neutral with regard to jurisdictional claims in published maps and institutional affiliations.

## Supplementary Material

Supplementary Information

## Figures and Tables

**Table 1:** Values of the fitting parameters of the volumetric EOS found by fitting the measured specific volumes to Eq. (12) with Eqs (13) and (14).

LC system (phase)	T_0_ (K)	A_0_ (cm^3^/g)	A_1_/10^−4^ (cm^3^/g/K)	A_2_/10^−6^ (cm^3^/g/K^2^)	B(T_0_, p_0_) (MPa)	b_2_/10^−3^(K^−1^)	γ_EOS_	Adj.-R^2^ coeff.
6BT (Cr E)	250.75	0.9038 ± 0.0001	3.62 ± 0.03	0.58 ± 0.03	2983 ± 10	2.62 ± 0.06	3.97 ± 0.08	0.9986
8BT (Cr E)	301.5	1.0027 ± 0.0003	8.31 ± 0.11	0.22 ± 0.02	2384 ± 22	3.74 ± 0.23	7.82 ± 0.27	0.9997
7CB (N)	302.0	0.9983 ± 0.0005	9.57 ± 0.43	10.2 ± 2.2	1704 ± 60	18.5 ± 2.9	13.99 ± 0.64	0.9992
7PCH (N)	303.3	1.0493 ± 0.0002	9.23 ± 0.12	3.21 ± 0.35	1755 ± 16	9.71 ± 0.45	10.50 ± 0.16	0.9999
8PCH (N)	309.13	1.0609 ± 0.0007	10.5 ± 0.5	4.16 ± 0.03	1592 ± 65	12.2 ± 2.7	12.03 ± 0.74	0.9986
8OCB (N)	342.0	1.0052 ± 0.0001	9.28 ± 0.07	2.33 ± 0.14	1731 ± 6	9.76 ± 0.28	11.37 ± 0.10	0.9996

The reference state is fixed at the phase transition temperature T_0_ at ambient pressure p_0_ = 0.1 MPa.

**Table 2:** Values of the fitting parameters of the MYEGA model (Eq. (5)) established for longitudinal relaxation times of the examined LC systems.

LC system (phase)	τ_0_ (s)	A_M_ (Kcm^3γ^/g^γ^)	D_M_/10^4^ (Kcm^3γ^/g^γ^)	γ_M_	Adj.-R^2^ coeff.
6BT (Cr E)	(3.25 ± 0.48)×10^−15^	−55 ± 4	0.78 ± 0.03	2.74 ± 0.03	0.9966
8BT (Cr E)	(2.60 ± 0.22)×10^−12^	−66 ± 3	0.61 ± 0.06	4.59 ± 0.03	0.9990
7CB (N)	(6.49 ± 0.40) ×10^−25^	−209 ± 16	2.35 ± 0.45	3.42 ± 0.05	0.9941
7PCH (N)	(3.59 ± 0.41) ×10^−19^	−204 ± 8	1.70 ± 0.10	3.96 ± 0.02	0.9968
8PCH (N)	(9.92 ± 0.11) ×10^−30^	−294 ± 5	4.15 ± 0.24	3.53 ± 0.02	0.9961
8OCB (N)	(2.29 ± 0.27) ×10^−19^	−196 ± 6	1.53 ± 0.09	4.13 ± 0.03	0.9983

**Table 3:** Values of the fitting parameters of the activation volumetric EOS by fitting the activation volumes evaluated using the MYEGA model to Eq. (9) with Eqs (10) and (11).

LC system (phase)	F_0_(cm^3^/mol)	F_1_(cm^3^/mol/K)	F_2_/10^#x02212;4^(cm^3^/mol/K^2^)	B_act_(T_0_, p_0_) (MPa)	g_2_/10^−3^(K^−1^)	γ_act_	γ_act_ from Eq. (19)
6BT (Cr E)	42.97 ± 0.11	0.1871 ± 0.0003	0.00 ± 0.15	998.9 ± 5.6	0.214 ± 0.067	0.981 ± 0.023	0.982
8BT (Cr E)	61.38 ± 0.01	0.2090 ± 0.0002	−6.11 ± 0.05	432.92 ± 0.21	0.409 ± 0.014	1.109 ± 0.004	1.105
7CB (N)	61.27 ± 0.50	2.138 ± 0.056	26.1 ± 1.8	73.69 ± 0.36	4.42 ± 0.38	0.518 ± 0.007	0.512
7PCH (N)	66.59 ± 0.06	1.315 ± 0.007	35.7 ± 2.2	121.22 ± 0.25	1.89 ± 0.13	0.59 ± 0.01	0.59
8PCH (N)	66.41 ± 0.18	2.386 ± 0.017	24.1 ± 3.1	67.72 ± 0.20	0	0.363 ± 0.003	0.367
8OCB (N)	74.98 ± 0.07	1.392 ± 0.007	21.5 ± 1.2	113.75 ± 0.17	0	0.459 ± 0.002	0.459

The reference state is fixed at the phase transition temperature T_0_ (shown in Table 1) at ambient pressure p_0_ = 0.1 MPa. The fitted value of γ_act_ is compared with that calculated from Eq. (19) for each material.
