# Supplementary material for: Activation volume of selected liquid crystals in the density scaling regime
Source: Sci Rep. 2017 Feb 9;7:42174. doi: 10.1038/srep42174 (PMC5299607; doi:10.1038/srep42174)
Supplement: Supplementary Information [file srep42174-s1.pdf]

Activation volume of selected liquid crystals in the density scaling regime

A. Grzybowski,[1,2,*] S. Urban,[3] S. Mroz,[1,2] and M. Paluch[1,2]

[1] *Institute of Physics, University of Silesia, ul. Uniwersytecka 4, 40-007 Katowice, Poland*

[2] *Silesian Center for Education and Interdisciplinary Research, ul. 75 Pułku Piechoty 1a, 41-500 Chorzów, Poland*

[3] *Institute of Physics, Jagiellonian University, S. Łojasiewicza 11, 30-348 Kraków, Poland*

* Corresponding author's e-mail: andrzej.grzybowski@us.edu.pl

Supplementary Information

S1. Model-dependent relations between parameters of the volumetric and activation volumetric equations of state

To conduct a comparative study of the activation volumetric properties of the selected LC systems in terms of the Avramov and MYEGA models, we have made an attempt to formulate the relationships relied on the T-V MYEGA models between the exponents γ_{EOS} and γ_{act} as well as between the isothermal bulk modulus B_{act} and B_T. It has turned out that such a derivation is only possible in an approximate way due to the morphology of the T-V MYEGA equation (Eq. (5) in the main part),

$$\tau_{||} = g_M(T,V) = \tau_{0_M} \exp\left[\frac{D_M}{TV^{\gamma_M}} \exp\left(\frac{A_M}{TV^{\gamma_M}}\right)\right] \quad \text{(S1)}$$

which does not allows us to arrive directly at a formula for the exponent γ_{act} in the activation volumetric EOS (Eq. (9) in the main part)

$$\left(\frac{V_{act}(T,p_0)}{V_{act}(T,p)}\right)^{\gamma_{act}} = 1 + \frac{\gamma_{act}}{B_{act}(T,p_0)}(p - p_0) \ . \quad \text{(S2)}$$

Similarly to the method applied to formulate Eq. (17) in the main part, i.e., the relation, $\gamma_{act} = \gamma_{EOS}/(\gamma_{EOS} - \gamma D)$, in terms of the T-V Avramov model, we express the ratio $V_{act}(T,p_0)/V_{act}(T,p)$ in Eq. (S2) herein by using the T-V MYEGA model. As a result, from Eq. (6) with Eq. (8) in the main part, i.e., $V_{act} = RT(\partial \ln g_{\mathrm{mod}}(\Gamma)/\partial\Gamma)\gamma\Gamma / B_T(p)$ with $(\partial \ln g_M(\Gamma)/\partial\Gamma) = D_M(1 + A_M\Gamma)\exp(A_M\Gamma)$, we find

$$\frac{V_{act}(T,p_0)}{V_{act}(T,p)} = \frac{(1 + A_M\Gamma_0)\Gamma_0 B_T(p)}{(1 + A_M\Gamma)\Gamma B_T(p_0)} \exp[-A_M(\Gamma - \Gamma_0)] \quad \text{(S3)}$$

where $\Gamma = h(\rho)/T$ and $\Gamma_0 = h(\rho(T,p_0))/T$ in the GDS regime. To establish a relationship between $V_{act}(T,p_0)/V_{act}(T,p)$ and the ratio $V(T,p_0)/V(T,p)$ in the volumetric EOS (Eq. (12) in the main part),

$$\left(\frac{V(T,p_0)}{V(T,p)}\right)^{\gamma_{EOS}} = 1 + \frac{\gamma_{EOS}}{B_T(p_0)}(p - p_0) \tag{S4}$$

one can employ the first-order Taylor series expansion of the quotient $(1 + A_M\Gamma_0)/(1 + A_M\Gamma) \cong 1 - A_M(\Gamma - \Gamma_0)/(1 + A_M\Gamma_0)$ about $\Gamma = \Gamma_0$ in Eq. (S3). Since one may also apply the first-order Taylor series expansion to the exponent $\exp[-A_M(\Gamma - \Gamma_0)/(1 + A_M\Gamma_0)] \cong 1 - A_M(\Gamma - \Gamma_0)/(1 + A_M\Gamma_0)$ about $\Gamma = \Gamma_0$, then Eq. (S3) can be approximately reduced to

$$V_{act}(T,p_0)/V_{act}(T,p) = (B_T(p)/B_T(p_0))(\Gamma/\Gamma_0)^{-1}\exp[-A_M\Gamma_0(\Gamma/\Gamma_0 - 1)(2 + A_M\Gamma_0)/(1 + A_M\Gamma_0)].$$

The exponent in the latter equation can be transformed to a power form by the next first-order Taylor series expansion as follows

$$[\exp(\Gamma/\Gamma_0 - 1)]^{-A_M\Gamma_0(2+A_M\Gamma_0)/(1+A_M\Gamma_0)} \cong [1 + \Gamma_0^{-1}(\Gamma - \Gamma_0)]^{-A_M\Gamma_0(2+A_M\Gamma_0)/(1+A_M\Gamma_0)}.$$

Consequently, $V_{act}(T,p_0)/V_{act}(T,p) = (B_T(p)/B_T(p_0))(\Gamma/\Gamma_0)^{-1-A_M\Gamma_0(2+A_M\Gamma_0)/(1+A_M\Gamma_0)}$. From the volumetric EOS (S4), $B_T(p)/B_T(p_0) = (V(T,p_0)/V(T,p))^{\gamma_{EOS}}$, which can be easily explained by Eq. (16) in the main part,

$$B_T(p) = B_T(p_0) + \gamma_{EOS}(p - p_0). \tag{S5}$$

If the PDS law is obeyed, then $\Gamma/\Gamma_0 = (V(T,p_0)/V(T,p))^{\gamma}$. In this case, we arrive at an isothermal auxiliary equation

$$\left(\frac{V_{act}(T,p_0)}{V_{act}(T,p)}\right)^{\gamma_{EOS}/(\gamma_{EOS} - \gamma[1 + A_M\Gamma_0(2+A_M\Gamma_0)/(1+A_M\Gamma_0)])} = 1 + \frac{\gamma_{EOS}}{B_T(p_0)}(p - p_0). \tag{S6}$$

Calculating the isothermal bulk modulus for the activation volume from Eq. (S6) and comparing the obtained result with the linear pressure dependence of $B_{act}(T,p)$ given by Eq. (15) in the main part,

$$B_{act}(T,p) = B_{act}(T,p_0) + \gamma_{act}(p - p_0) \tag{S7}$$

we find the approximate equations for γ_{act} and $B_{act}(T, p_0)$, which depend on the parameter A_M of the MYEGA model (S1),

$$\gamma_{act} = \frac{\gamma_{EOS}}{\gamma_{EOS} - \gamma\left[1 + A_M \Gamma_0 (2 + A_M \Gamma_0)/(1 + A_M \Gamma_0)\right]} \tag{S8}$$

$$B_{act}(T, p_0) = \frac{B_T(p_0)}{\gamma_{EOS} - \gamma\left[1 + A_M \Gamma_0 (2 + A_M \Gamma_0)/(1 + A_M \Gamma_0)\right]} \tag{S9}$$

where $\Gamma_0 = T^{-1} V^{-\gamma}(T, p_0)$ is constant at T=*const*. Since one can expect similarly to γ_{EOS} that $\gamma_{act} = \left(\partial B_{act}(p,T)/\partial p\right)_T \cong const$ at different temperatures, the value of the parameter Γ_0 should remain unchanged for a given material at least to a good approximation.

S2. Results of the analysis based on the T-V Avramov model

As an example, in Fig. S1, we present details of the analysis based on the T-V Avramov model. The dielectric isotherms of 8BT (Fig. S1(a)) measured as a function $\tau_{||}$(T,p) have been transformed to its T-V representation (not shown herein) by using the volumetric EOS (shown in detail as Eq. (12) with Eqs. (13) and (14) in the main part and in its isothermal form as Eq. (S4) in this document) fitted to the pVT experimental data for 8BT (Fig. S1(b)), making the assumption that the reference state (T_0,p_0) in the volumetric EOS is fixed at the crystal – Cr E transition temperature T_0=301.5K at ambient pressure p_0=0.1MPa. Then, we can directly describe the dependence $\tau_{||}$(T,p) as shown in Fig. S1(a) by using Eq. (4),

$$\tau_{||} = g_A(T,V) = \tau_0 \exp\left[\left(\frac{A}{TV^{\gamma}}\right)^D\right] \tag{S10}$$

with the specific volume V(T,p) expressed by the volumetric EOS with the values of its parameters found by fitting pVT measurement data to this EOS (see Fig. S1(b) in this document and Table 1 in the main part). A good quality of the fitting procedure to Eq. (S10) is confirmed by the adjusted measure R^2=0.9997 for this LC system (see Table S1 for other

tested LCs) and also reflected in the density scaling of the longitudinal relaxation times of 8BT, which is well satisfied (see Fig. S1(c)) with the value of the scaling exponent, γ=4.59±0.03, evaluated by fitting to the Avramov model (Eq. (S10)). Subsequently, we have established (Fig. S1(d)) the activation volumes V_{act} from Eq. (6) with Eq. (7) in the main part, i.e., $V_{act} = RT\left(\partial \ln g_{\mathrm{mod}}(\Gamma)/\partial\Gamma\right)\gamma\Gamma / B_T(p)$ with $\left(\partial \ln g_A(\Gamma)/\partial\Gamma\right) = DA^D\Gamma^{D-1}$, at temperatures and pressures (T,p) at which the dependences $\tau_{||}$(T,p) have been determined. The isothermal dependences V_{act}(T,p) for 8BT at several different temperatures have been fitted to the activation volumetric EOS (shown in detail as Eq. (9) with Eqs. (10) and (11) in the main part and in its isothermal form as Eq. (S2) in this document), taking the same reference state (T_0,p_0) as that assumed to parametrize the pVT data for this LC system by means of the volumetric EOS. In this way, for all the examined LC systems, we have found the values of the parameters of the T-V Avramov model, and the activation volumetric and volumetric EOSs, given as Eqs. (4), (9)-(11), and (12)-(14) in the main document, which are collected respectively in Tables S1 and S2 in this document and Table 1 in the main document. Based on the activation volume analysis, we have determined the isothermal bulk moduli for V_{act}. The isothermal pressure dependences of B_{act} are shown in Fig. S2 for 8BT in the Cr E phase and 7CB in the nematic phase as examples.

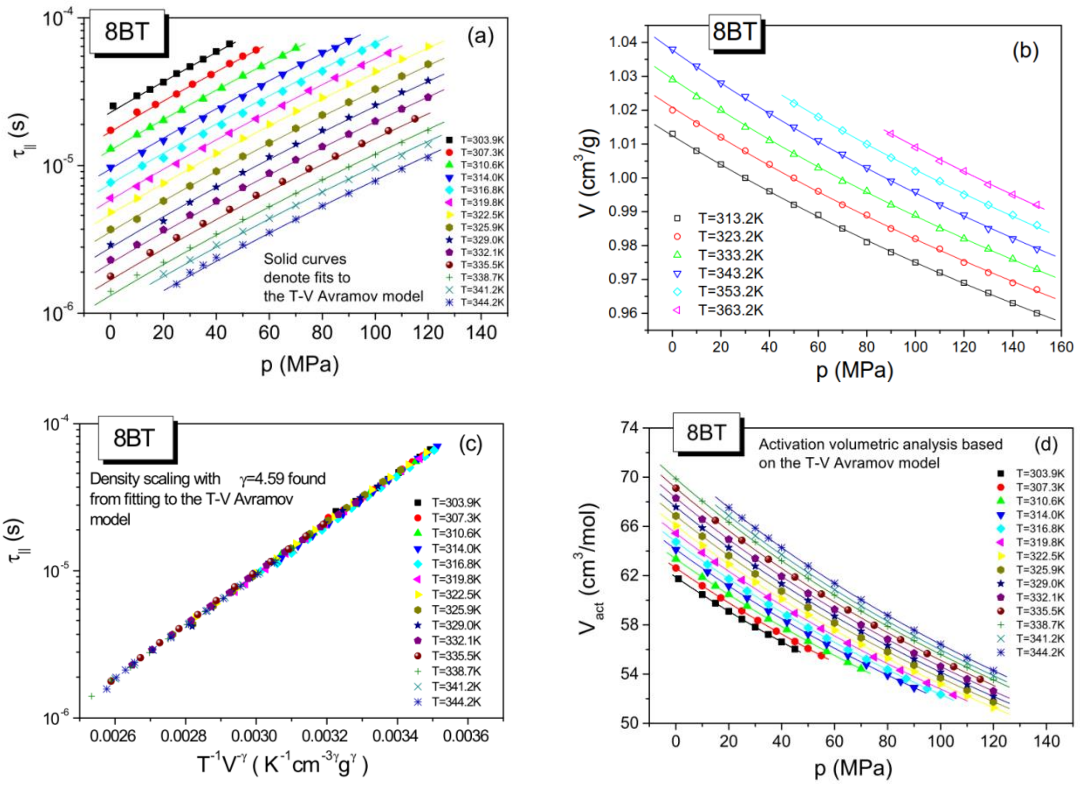

Fig. S1

The analysis of the activation volumes of 8BT in terms of the T-V Avramov model. (a) Pressure dependences of experimental longitudinal relaxation times along different isotherms and their fits to Eq. (4) (see Table S1 for the values of its parameters) with the specific volume expressed as the function V(T,p) by the volumetric EOS given by Eq. (12) with Eqs. (13) and (14) in the main part. (b) Pressure dependences of specific volumes measured along different isotherms and their fits to the volumetric EOS (see Table 1 in the main part for the values of its parameters). (c) Plot of the master plot according to the power law density scaling law obeyed by longitudinal relaxation times. (d) Pressure dependences of the activation volumes evaluated from Eq. (6) with Eq. (7) in the main part and their fits to the activation volumetric EOS given by Eq. (9) with Eqs. (10) and (11) in the main document (see Table S2 for the values of its parameters).

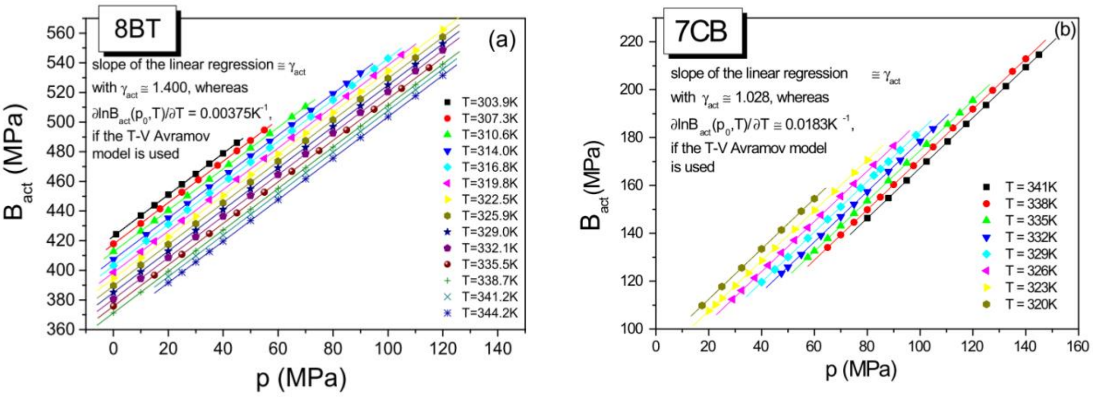

Fig. S2

Plot of the pressure dependences of the isothermal bulk moduli for the activation volumes evaluated for 8BT and 7CB in terms of the T-V Avramov model (panels (a) and (b), respectively). The solid lines denote the linear dependences that are in accord with Eq. (11) in the main part (shown as Eq. (S7) in this document), which complies with the activation volumetric EOS given by Eq. (9) with Eqs. (10) and (11) in the main part (its isothermal form is shown as Eq. (S2) in this document). See Table S2 in this document for the activation volumetric EOS parameters established for the values of V_{act} evaluated based on the T-V Avramov model.

Table S1. Values of the fitting parameters of the Avramov model (Eq. (S10) in this document and Eq. (4) in the main part) established for longitudinal relaxation times of the examined LC systems

LC system (phase)	τ_0 (s)	$A/10^4$ ($Kcm^{3\gamma}/g^{\gamma}$)	D	γ	Adj.-R^2 coeff.
6BT (Cr E)	$(2.64\pm0.18)\times10^{-17}$	4.47±0.41	0.66±0.06	2.74±0.03	0.9966
8BT (Cr E)	$(1.33\pm0.36)\times10^{-16}$	24.6±1.5	0.49±0.03	4.59±0.03	0.9990
7CB (N)	$(2.22\pm0.26)\times10^{-52}$	$(5.88\pm0.77)\times10^{14}$	0.12±0.01	3.43±0.05	0.9935
7PCH (N)	$(3.52\pm0.86)\times10^{-52}$	$(8.24\pm0.44)\times10^{15}$	0.12±0.01	3.97±0.02	0.9964
8PCH (N)	$(3.01\pm0.32)\times10^{-85}$	$(3.31\pm0.90)\times10^{28}$	0.08±0.03	3.50±0.03	0.9929
8OCB (N)	$(8.14\pm0.51)\times10^{-75}$	$(9.13\pm0.72)\times10^{31}$	0.07±0.01	4.16±0.04	0.9975

Table S2. Values of the fitting parameters of the activation volumetric EOS found by fitting the activation volumes evaluated using the Avramov model to Eq. (9) with Eqs. (10) and (11) in the main part. The reference state in the EOS is fixed at the phase transition temperature T_0 (shown in Table 1 in the main part) at ambient pressure p_0=0.1MPa. The fitted value of γ_{act} is compared with that calculated for each material from Eq. (17) in the main part, i.e., from the relation, $\gamma_{act} = \gamma_{EOS} / (\gamma_{EOS} - \gamma D)$.

LC system (phase)	F_0 (cm^3/mol)	F_1 (cm^3/mol/K)	$F_2 /10^{-4}$ ($cm^3/mol/K^2$)	$B_{act}(T_0,p_0)$ (MPa)	$g_2/10^{-3}$ (K^{-1})	γ_{act}	γ_{act} from Eq.(17)
6BT (Cr E)	45.04±0.01	0.1469±0.0001	0.816±0.001	1371.81±0.01	2.62±0.01	1.825±0.001	1.825
8BT (Cr E)	61.33±0.01	0.2197±0.0001	2.597±0.006	426.92±0.03	3.75±0.01	1.400±0.001	1.400
7CB (N)	66.17±0.13	1.077±0.016	224.7±5.4	124.82±0.43	18.3±0.3	1.028±0.004	1.031
7PCH (N)	68.25±0.04	0.7888±0.0049	55.8±1.6	170.39±0.40	8.35±0.14	1.006±0.006	1.046
8PCH (N)	76.29±0.04	1.0720±0.0054	101.1±2.1	132.81±0.26	11.1±0.2	1.002±0.004	1.022
8OCB (N)	68.22±0.01	0.7826±0.0010	60.86±0.47	157.65±0.07	9.75±0.04	1.035±0.002	1.024

S3. Comparison of the density scaling behavior evaluated in terms of the T-V Avramov and MYEGA models

In Fig. S3, the fitted curves generated from the Avramov and MYEGA density scaling models are compared as functions of the scaling variable $\Gamma=T^{-1}V^{-\gamma}$. This comparison shows that the curves fitted to the MYEGA model reveal a stronger negative curvature than those generated from the Avramov model, especially in case of the LC systems investigated in the nematic phase (Fig. S3(b)). Since the fitted values of the scaling exponents γ and γ_M are nearly the same for a given tested material, i.e., the scaling variables Γ determined from the Avramov and MYEGA model are only slightly different for this material, Fig. S3 reliably illustrates the small differences in the density scaling curves fitted to the Avramov and MYEGA model, which are caused by the other parameters of the models except for the scaling exponent. For instance, one can notice that the slower convergence and the weaker negative curvature of the

curves fitted to the Avramov model applied to the examined LC systems in the nematic phase are additionally associated with the extremely small values of the preexponential factor τ_0, which are physically much more reasonable in case of the MYEGA model, although the latter model easier and better reflects the negative curvature of the dependences $\log_{10}\tau_{||}(\Gamma)$. Nevertheless, the preexponential factor τ_0 does not affect the activation volume defined by Eq. (3) in the main part via the derivative of $\ln\tau_{||}$ with respect to pressure. Thus, we need to look deeper into what determines the activation volume in both the Avramov and MYEGA models, which is done in the section *Results and Discussion* in the main part.

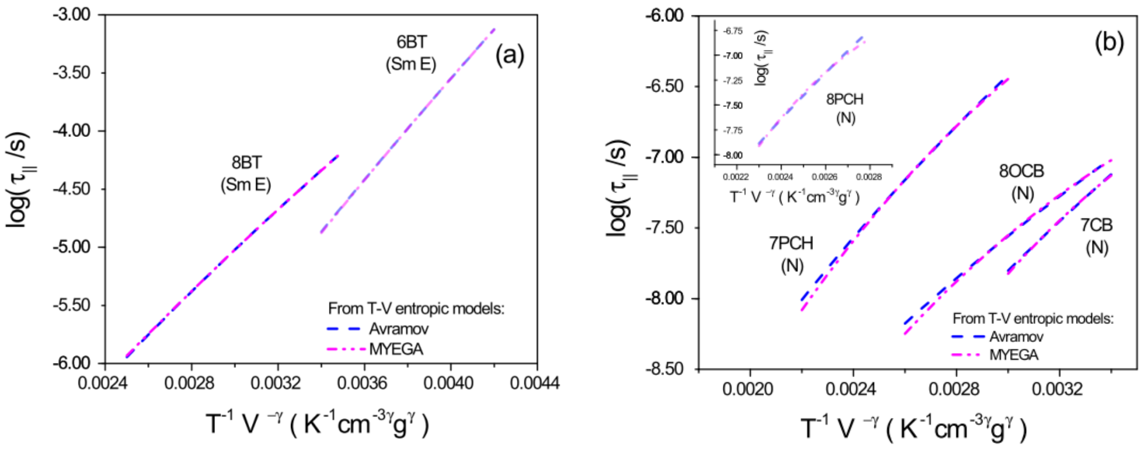

Fig. S3

Plot of the fitted curves in terms of the T-V Avramov and MYEGA models (i.e., Eqs. (4) and (5) in the main part, also shown in this document as Eqs. (S10) and (S1)) with the values of their parameters collected respectively in Tables S1 in this document and Table 2 in the main part for all the LC systems examined in the Cr E phase (a) and in the nematic phase (b) in the dielectric measurement ranges.

S4. Physical meaning of the fitting parameters in the T-V Avramov and MYEGA models in case of GF systems

It is worth noting that not only the density scaling exponent but also the other fitting parameters have well-defined physical meanings in both the entropic models. The preexponential factor in each model is typically interpreted as a high-temperature limiting value of the considered relaxation times. However, the parameters *A*, *D*, A_M, and D_M result from specific assumptions underlying the entropic models.

In the T-V Avramov model, the parameter *A* has been argued[27] to involve the maximal energy barrier $E_{\max}(T_r,V_r)$ and the energy barrier distribution width $\sigma(T_r,V_r)$ for the considered relaxation process at a reference state (T_r,V_r), i.e.,

$$A = \left(E_{\max}(T_r,V_r)/\sigma(T_r,V_r)\right)^{1/D} T_r V_r^{\gamma} \ . \tag{S11}$$

In case of GF, the reference state (T_r,V_r) is usually defined at the glass transition at ambient pressure, although one can choose another reference state, e.g., the melting point at ambient pressure. In case of LC systems, the reference state can be assumed at the transition to another LC phase at ambient pressure. The reference parameters $E_{\max}(T_r,V_r)$ and $\sigma(T_r,V_r)$ are embedded respectively in the volume-dependent maximal energy barrier for the considered relaxation process,[27]

$$E_{\max}(V) \approx E_{\max}(T_r,V_r)(V_r/V)^{D(\gamma-\gamma_G)}, \tag{S12}$$

and the total system entropy,[26]

$$S(T,V) \approx S(T_r,V_r) + 0.5ZR\ln\left(\sigma(T,V)/\sigma(T_r,V_r)\right) \tag{S13}$$

with the temperature-volume dependent energy barrier distribution width

$$\sigma(T,V) = \sigma(T_r,V_r)[(V/V_r)^{\gamma_G} T/T_r]^D, \tag{S14}$$

where *R* is the gas constant, and the Grüneisen parameter

$$\gamma_G = V\alpha_p C_V^{-1}\kappa_T^{-1} \tag{S15}$$

can be evaluated for a supercooled liquid near the glass transition from the isobaric thermal expansivity α_p, the isothermal compressibility κ_T, and the isochoric heat capacity C_V, whereas

Z has been interpreted to be proportional to the number of available pathways for local motions of a molecule or polymer segment (and this number has been also assumed to be roughly proportional to the coordination number of the liquid lattice),[26,29] The parameter D comes from the original derivation[26] of Eq. (S10), i.e., Eq. (4) in the main part, which results in

$$D = 2C_V Z^{-1} R^{-1}. \qquad \text{(S16)}$$

In the T-V MYEGA model, the fitting parameters A_M and D_M are related respectively to some volume-dependent energetic parameters,[23]

$$B_M(V) \cong B(V_r)(V_r/V)^{\gamma} \qquad \text{(S17)}$$

and

$$H_M(V) = H_M(V_r)(V_r/V)^{\gamma}, \qquad \text{(S18)}$$

where $B_M(V)$ is an effective activation barrier for the considered relaxation process and $H_M(V)$ is the energy difference between two states (on the assumption that network constraints in a two-state simple system may be intact or broken).[23,32] The detailed equations,

$$D_M = B_M(V_r)V_r^{\gamma}/(3Nk_B \ln\Omega) \qquad \text{(S19)}$$

and

$$A_M = H_M(V_r)V_r^{\gamma}/k_B, \qquad \text{(S20)}$$

involve the reference parameters $B_M(V_r)$ and $H_M(V_r)$, the Boltzmann constant k_B, the number of atoms N, and the number of degenerate configurations Ω per floppy mode.

In the original Adam-Gibbs model,[33] the parameter B_M was assumed to be a constant dependent on the product of the critical configurational entropy s_c^* (i.e., the configurational entropy of the smallest cooperative subsystem that can perform a rearrangement into another configuration) and the potential energy barrier $\Delta\mu$ hindering the transition to a new configuration, where $\Delta\mu$ is related to a difference in the Gibbs free energies of the

rearrangeable subsystems and the others. However, further investigations have shown[21,23,25] that the dependence of the parameter B_M on thermodynamic conditions cannot be neglected.

For our discussion (presented in the main part) on two entropic models applied to study the density scaling in LC systems, it is important that the parameter A_M is embedded in the equation formulated for the configurational entropy within the T-V MYEGA model as[23]

$$S_c(T,V) = 3Nk \ln \Omega \exp(-A_M T^{-1} V^{-\gamma}), \quad \text{(S21)}$$

which can be also expressed as follows

$$S_c(T,V) = 3Nk \ln \Omega \exp(-H_M(V) k_B^{-1} T^{-1}). \quad \text{(S22)}$$

References cited in Section S4 (*keeping their original numbers used in the main part*)

[21] Masiewicz, E., Grzybowski, A., Grzybowska, K., Pawlus, S., Pionteck, J. & Paluch, M. Adam-Gibbs model in the density scaling regime and its implications for the configurational entropy scaling. *Sci. Rep.* **5**, 13998-1 - 13998-13 (2015).

[23] Masiewicz, E., Grzybowski, A., Sokolov, A. P. & Paluch, M. Temperature-Volume Entropic Model for Viscosities and Structural Relaxation Times of Glass Formers. *J. Phys. Chem. Lett.* **3**, 2643-2648 (2012).

[25] Alba-Simionesco, C. & Tarjus, G. Temperature versus density effects in glassforming liquids and polymers: A scaling hypothesis and its consequences. *J. Non-Cryst. Solids* **352**, 4888 - 4894 (2006).

[26] Casalini, R., Mohanty, U., & Roland, C. M. Thermodynamic interpretation of the scaling of the dynamics of supercooled liquids. *J. Chem. Phys.* **125**, 014505-1 - 014505-9 (2006).

[27] Grzybowski, A., Paluch, M., Grzybowska, K. & Haracz, S. Communication: Relationships between Intermolecular potential, thermodynamics, and dynamic scaling in viscous systems. *J. Chem. Phys.* **133**, 161101-1 - 161101-4 (2010).

[29] Avramov, I., & Milchev, A. Effect of Disorder on Diffusion and Viscosity in Condensed Systems. *J. Non-Cryst. Solids* **104**, 253-260 (1988).

[32] Mauro, J. C., Yue, Y. Z., Ellison, A. J., Gupta, P. K. & Allan, D. C. Viscosity of glass-forming liquids. *Proc. Natl. Acad. Sci. U.S.A.* **106**, 19780 - 19784 (2009).

[33] Adam, G. & Gibbs, J. H. On the Temperature Dependence of Cooperative Relaxation Properties in Glass-Forming Liquids. *J. Chem. Phys.* **43**, 139-146 (1965).
